# Elevated salivary C-reactive protein predicted by low cardio-respiratory fitness and being overweight in African children

**DOI:** 10.5830/CVJA-2012-058

**Published:** 2012-10

**Authors:** T Naidoo, K Konkol, AJ Mckune, B Biccard, K Dubose

**Affiliations:** Department of Biokinetics, Exercise and Leisure Sciences, School of Health Sciences, University of KwaZulu-Natal, Durban, South Africa; Department of Biokinetics, Exercise and Leisure Sciences, School of Health Sciences, University of KwaZulu-Natal, Durban, South Africa; Department of Biokinetics, Exercise and Leisure Sciences, School of Health Sciences, University of KwaZulu-Natal, Durban, South Africa; Perioperative Research Group, Department of Anaesthetics, Nelson R Mandela School of Medicine, University of KwaZulu-Natal, Durban, South Africa; Physical Activity Promotion Laboratory, Department of Kinesiology, East Carolina University, USA

**Keywords:** inflammation, C-reactive protein, body composition, children, cardio-respiratory fitness

## Abstract

**Introduction:**

C-reactive protein (CRP) is a sensitive marker of systemic inflammation and is an independent risk factor for cardiovascular disease. The aim of the study was to examine the relationship between salivary CRP, cardio-respiratory fitness and body composition in a paediatric population.

**Methods:**

This was a cross-sectional study of 170 black South African children (age 9.41 ± 1.55 years, 100 females, 70 males) in grades 3 to 7. Unstimulated whole saliva samples were obtained for the analysis of CRP. Height, mass, skin-fold thickness, resting blood pressure, and waist and hip circumference measurements were obtained. Cardio-respiratory fitness was assessed using a 20-m multi-stage shuttle run. Children were classified as overweight/obese according to the Center for Disease Control and Prevention (CDC) body mass index (BMI) percentile ranking, and meeting percentage body fat recommendations, if percentage body fat was ≤ 25% in boys and ≤ 32% in girls. The cut-off point for low cardio-respiratory fitness was a predicted aerobic capacity value ≤ the 50th percentile for the group. Contributions of low cardio-respiratory fitness, overweight/obesity, and not meeting percentage body fat recommendations, to elevated salivary CRP (≥ 75th percentile) concentration and secretion rate were examined using binary logistic regression analysis with a backward stepwise selection technique based on likelihood ratios.

**Results:**

Poor cardio-respiratory fitness was independently associated with elevated salivary CRP concentration (OR 3.9, 95% CI: 1.7–8.9, *p* = 0.001). Poor cardio-respiratory fitness (OR 2.7, 95% CI: 1.2–6.1, *p* = 0.02) and overweight/obesity (BMI ≥ 85th percentile) (OR 2.5, 95% CI: 1.1–5.9, *p* = 0.03) were independent predictors of elevated salivary CRP secretion rate.

**Conclusion:**

The results suggest a strong association between poor cardio-respiratory fitness and/or overweight/obesity and inflammatory status in children, based on elevated salivary CRP levels.

## Abstract

Inflammation has been hypothesised as a potential mediator of the association between obesity, physical inactivity and the development of chronic diseases, such as cardiovascular disease (CVD), type 2 diabetes, depression and cancer.^1^ Low-grade systemic inflammation, a condition marked by a chronic two-to three-fold increase in circulating inflammatory markers,^2^ is considered important in the chronic disease process through promoting the development of insulin resistance, atherosclerosis, neurodegeneration, and tumour growth.^3^ Systemic C-reactive protein (CRP) is a sensitive marker of low-grade systemic inflammation and is an independent risk factor for CVD.^4^-^6^ It is therefore important to characterise the determinants of individual differences in inflammatory markers such as CRP.^7^

The current worldwide pandemic of childhood obesity and low levels of cardio-respiratory fitness (CRF) makes the understanding of inflammation and interventions to reduce inflammation, important areas of research.^8^,^9^ Obese children have higher levels of systemic CRP compared to children of normal weight, suggesting that chronic low-grade systemic inflammation is an early complication of childhood obesity.^8^ In addition, elevated systemic CRP in young children is a risk factor for the development of the metabolic syndrome.^9^

Research has revealed an association between elevated systemic CRP and low CRF.^10^ By contrast, several studies show that systemic CRP is lowered by a reduction in visceral fat mass and/or increased physical activity or exercise.^11^,^12^ In healthy, pre-pubertal children, elevated CRF was shown to protect against low-grade inflammation.^10^

There is limited research in this area in Africa, although a recent study of black South African adolescents reported a trend for increased levels of serum CRP in individuals with a higher percentage body fat.^13^ The study also found a negative correlation between fitness levels and serum CRP.^13^

However, the investigation of inflammatory levels is delayed by the lack of non-invasive methods to assess inflammation that would enable research in large representative populations and in children.^7^ The investigation of inflammatory biomarker levels are commonly measured in blood collected through venipuncture. However, venipuncture is an invasive procedure and requires skilled professionals, laboratory equipment and considerable financial resources.^7^ By contrast, saliva collection is non-invasive, stress- and pain-free and constitutes an alternative strategy to prospectively assess immune activity in large samples.^7^

The measurement of biomarkers in saliva to examine numerous clinical conditions has increased over the last decade.^14^,^15^ Recently, a high-sensitivity commercially available enzyme-linked immunoassay (ELISA) adapted to measure CRP in human saliva was validated against serum CRP.^7^

The findings provided initial support for the use of salivary CRP as an alternative marker of inflammation, with a moderate-to-strong association (*r* = 0.72, *p* < 0.001) between CRP measured in saliva and serum.^7^ In addition, salivary and plasma CRP were shown to be moderately correlated (*r* = 0.61) in females, and salivary CRP was able to discriminate between high and low levels of plasma CRP, using a clinically relevant cut-off point of 3 mg/l.^16^ These results were consistent with strong correlations between saliva and serum CRP levels in earlier animal studies, specifically in pigs (*r* = 0.73),^17^ and in healthy and diseased dogs (*r* = 0.87 and *r* = 0.84, respectively).^18^

In addition to being non-invasive, the assessment of CRP in the saliva allows data collection to take place in the participant’s natural environments (home and school). Furthermore, CRP in saliva is stable at room temperature for up to eight hours after collection, making the sampling of saliva outside of research facilities a viable option.^7^ Previous studies have also shown that serum CRP does not have a circadian rhythm^19^,^20^ and therefore saliva sampling aimed to measure salivary CRP does not require one to follow a standardised time-collection schedule to avoid diurnal variations.^7^

Recently, salivary CRP was shown to be a good measure of discrimination for the clinically relevant serum CRP cut-off point in adults.^7^ Participants with high salivary CRP levels were more likely to have higher IL-6 levels and body mass index (BMI) and to smoke, compared to participants with low salivary CRP levels.^7^ The results suggested that salivary CRP may represent an alternative marker of cardiovascular risk in adults,^7^ however the association with a major risk factor for the development of chronic diseases, low CRF,^21^ was not included in the analysis.

There is limited research on the relationship between body composition, CRF and salivary CRP in adults and specifically in paediatric populations. The aim of this study was to examine the relationship between salivary CRP, body composition and cardio-respiratory variables in grade 3 to 7 children.

## Methods

One hundred and seventy black South African children (100 females, 70 males) in grades 3 to 7 (age 9.41 ± 1.55 years) participated in the study. Participants were recruited from an urban, combined junior and senior primary school in Pietermaritzburg, KwaZulu-Natal. Gate-keeper permission to perform the study was obtained from the KwaZulu-Natal Department of Education, the school’s headmaster and governing body. The study was approved by the institution’s Biomedical Ethics Research Committee.

Once permission to continue was obtained, a meeting was held with parents/guardians and children to discuss the research details and expectations of the participants. Written informed consent was obtained at the meeting.

The guardians/parents completed a medical history form that included sections on infectious, immune and salivary gland disorders. The parents/guardians were trained in the salivary collection procedure and were provided with instructions regarding brushing teeth and the intake of food and drink on the morning of the saliva collection. These standardised instructions are outlined below.

## Saliva collection and C-reactive protein analysis

Each grade was tested on a separate morning over a week, starting with grade 3 on Monday and ending with grade 7 on Friday. Saliva samples were collected between 07:30 and 08:30, approximately 90 minutes after waking.

The parents/guardians and children were requested to adhere as closely as possible to the following standardised saliva collection instructions (Salimetrics 2010): The children should (1) not eat a major meal (breakfast) within 60 minutes of sample collection, (2) not brush their teeth prior to sample collection (this may cause the gums to bleed causing blood contamination of the saliva), (3) avoid dairy products for 20 minutes before sample collection, (4) avoid foods with high sugar, acidity, or high caffeine content immediately before sample collection (these have all been shown to impact on the saliva pH, altering assay results), (5) rinse their mouths with water to remove food residue before sample collection, and swallow to increase hydration, and (6) wait at least 10 minutes after rinsing before collecting saliva to avoid sample dilution.

Upon arriving in the school hall at 07:00 the children sat for 20 minutes. Based on completion of a short health questionnaire and interview with the researchers upon arrival, no participant reported symptoms suggesting that he/she was sick (e.g. fever, flu, diarrhoea) and there were no reports of ‘bleeding gums’ or ‘tooth ache’ on the day of data collection.

Saliva samples were collected via unstimulated passive drool over a time period of five minutes. While seated, the children were asked to lean slightly forward, tilt their heads down and accumulate saliva in the floor of the mouth for a minute, which was subsequently swallowed. Following this, there was a four-minute collection where the children dribbled saliva through a 5-cm plastic straw into a pre-weighed polypropylene cryovial (5 ml capacity). Care was taken to allow saliva to dribble into the collecting tubes with minimal orofacial movement.

After collection, the cryovial was weighed in order to determine the saliva flow rate. The concentration of salivary CRP was expressed as the secretion rate of salivary CRP (pg/min) or the total amount of salivary CRP appearing on the mucosal surface per unit time. Salivary CRP secretion rate was calculated by multiplying absolute salivary CRP concentration (pg/ml) by saliva flow rate (ml/min). This latter value was calculated by dividing the total volume of saliva obtained in each sample (ml) by the time taken to produce each sample (4 min).

Samples were placed on dry ice immediately and kept frozen until reaching the laboratory, upon which they were stored at –70°C until analysis. Salivary CRP concentration (pg/ml) was determined in duplicate by the salivary C-reactive protein ELISA kit (Salimetrics, State College, PA, USA). The coefficients of variation (CV) of all duplicate samples were less than 20%.

## Body composition, cardiovascular and cardiorespiratory fitness

These measures were determined after saliva collection. Body composition was assessed by measuring height and weight to calculate BMI, skinfold thickness to predict body fat percentage, and waist and hip circumferences to calculate waist-to-hip ratios.

Height was measured to the nearest millimetre using a portable stadiometer (Nagata bw-1122h) and body mass was measured to the nearest 0.1 kg using a calibrated electronic scale (Nagata bw-1122h). Participants were asked to remove footwear and only wore their school physical education outfit that included shorts and a short-sleeve T-shirt.

Seated resting heart rate, measured to the nearest beat per minute (bpm). Resting blood pressure, measured to the nearest millimetre of mercury (mmHg) were recorded after a resting period of ten minutes.

Body fat percentage was determined using the four-site skinfold method.^22^ Triceps, biceps, supra-iliac and sub-scapular skinfolds were measured on the right side of the body using Harpenden© (West Sussex, UK: Quality Measurement, Ltd) skinfold calipers. Each site was measured twice to the nearest millimetre and the mean value was recorded. Circumferences at the waist (narrowest part of the torso) and hip (level of maximum extension of the buttocks) were measured to the nearest millimetre with a tape measure and the waist-to-hip ratio was calculated.^23^

CRF was assessed using the 20-m multi-stage shuttle run test that predicts an individual’s maximal aerobic capacity (VO_2max_).^24^ This test has been shown to be an appropriate predictor of CRF for the age groups participating in the study.^25^

## Statistical analysis

The Shapiro-Wilk algorithm was used to assess whether the body composition, cardiovascular and CRF variables, and salivary CRP concentration and secretion rates demonstrated a normal distribution. Both salivary CRP concentration and secretion rate were found to have skewness and kurtosis and so the values were log transformed before analysis.

Mean and standard deviations were calculated for the log-transformed CRP values as well as the demographic, body composition, cardiovascular and CRF variables. One-way ANOVA examined the differences in body composition, cardiovascular and CRF variables, and salivary CRP concentration and secretion rates by BMI categories. Tukey’s *post hoc* analysis was completed when appropriate.

Binary logistic regression was performed to determine the unadjusted and adjusted odds ratios for the prediction of elevated salivary CRP concentration and secretion rate by specific risk factors. The unadjusted odds ratio indicates the combined contribution of the three risk factors to elevated salivary CRP, while calculation of the adjusted odds ratio adjusts for the inter-relationships between them.

The choice of these risk factors was based on an *a priori* decision. The non-log transformed salivary CRP concentration and secretion rate data as well as the risk factors were divided into quartiles. The data for each child was then coded depending on whether they were positive or negative for the presence of each of the risk factors (defined below) and either positive or negative for the presence of a salivary CRP concentration or secretion rate ≥ 75th percentile.

Elevated salivary CRP was entered as the dependant variable with the risk factors entered into the model as covariates, using the enter method (unadjusted odd ratios) backward stepwise selection technique (adjusted odds ratios), based on likelihood ratios with entry and exit probabilities set to 0.05 and 0.1, respectively. The risk factors selected included the following:

• Not meeting percentage body fat recommendations of ≤ 25% for boys and ≤ 32% for girls. The body fat recommendations are based on the Fitnessgram Health Fitness Zone standards for body composition.^25^
• Being overweight/obese: ≥ 85th percentile for BMI, which was calculated as mass (kg) divided by height (m) squared. Growth charts have been published by the Center for Disease Control and Prevention (CDC) for BMI in boys and girls, two to 20 years old. These charts are percentiles showing the distribution of BMI at a given age and can be used to identify children who are at risk of being overweight (BMI > 85th percentile) or obese (BMI > 95th percentile).^25^ According to these CDC BMI-for-age standards, the participants were grouped into the following CDC BMI-for-age categories: normal weight (< 85th percentile), overweight (≥ 85th percentile to < 95th percentile), and obese (≥ 95th percentile).^26^ These cut-off points are unchanged from the 1998 expert committee recommendations and CDC and Institute of Medicine recommendations.^24^
• Demonstrating poor CRF (≤ 50th percentile) for predicted VO_2max_: ≤ 24.66 ml/kg/min. There are currently no standardised VO_2max_ data that can be used to categorise the CRF of children.^27^ However, for the present study, the authors divided the VO_2max_ data into quartiles and used VO_2max_ values ≤ 50th percentile to represent poor CRF in the children.

Statistical analysis was performed using SAS (version 9.2, Research Triangle, NC, USA) and SPSS 19.0 (SPSS) software. Significance level was set at *p* < 0.05.

## Results

Demographic, body composition, cardiovascular and CRF data and logged salivary CRP concentration and secretion rates for the children were divided according to three BMI categories (normal weight, overweight and obese). They are presented as means ± standard deviations.

The outcomes of the one-way ANOVAs examining the differences by BMI categories are indicated in Table 1. There were significant differences in age (F = 3.37, df = 2, 167, *p* = 0.037), height (F = 9.19, df = 2, 167, *p* < 0.001), mass (F = 127.52, df = 2, 167, *p* < 0.001), BMI (F = 248.89, df = 2, 167, *p* < 0.001), body fat percentage (F = 185.14, df = 2, 167, *p* = 0.001), systolic blood pressure (SBP) (F = 19.94, df = 2, 167, *p* < 0.001), diastolic blood pressure (DBP) (F = 23.95, df = 2, 167, *p* < 0.001), VO_2max_ (F = 17.76, df = 2, 167, *p* < 0.001), salivary CRP concentration (F = 5.89, df = 2, 167, *p* = 0.0034) and salivary CRP secretion rate (F = 5.90, df = 2, 167, *p* = 0.0033) between children of different BMI categories.

**Table 1.** Demographic, Body Composition, CRF Data For Normal Weight, Overweight And Obese Children. Values Are Mean ± Standard Deviation

	*Normal (n = 93) (males 38; females 55)*	*Overweight (n = 24) (males 8; females 16)*	*Obese (n =53) (males 24; females 29)*
Age (years)	9.71 (1.84)	9.25 (1.67)*	9.55 (1.63)
Height (cm)	137.12 (12.10)	137.75 (11.59)	140.75 (9.73)#
Mass (kg)	32.24 (7.68)**	37.90 (9.09)**	52.49 (13.68)**
BMI (kg/m^2^)	16.88 (1.50)**	19.64 (1.49)**	26.17 (5.03)**
Waist–hip ratio	0.77 (0.04)	0.79 (0.05)	0.83 (0.06)
Body fat (%)	19.39 (5.03)**	27.81 (5.02)**	36.42 (7.10)**
Resting HR (bpm)	85.96 (13.64)	84.54 (10.80)	85.57(13.53)
SBP (mmHg)	94.62 (11.99)	94.08 (7.63)	105.65 (12.06)^#^
DBP (mmHg)	62.25 (12.18)	62.67 (5.77)	72.55 (10.40)^#^
VO_2max_ (ml/kg/min)	28.61 (5.45)	25.02 (3.89)	22.72 (3.28)^#^
Salivary CRP (pg/ml)	6.77 (0.92)	6.85 (0.85)	7.31 (0.93)^&^
Salivary CRP (pg/min)	6.68 (0.98)	6.79 (0.75)	7.25 (0.99)^&^

*p < 0.05 for overweight vs normal weight; **p < 0.001 for all weight categories different from each other; ^#^*p* < 0.05 for obese vs normal weight and overweight; ^&^*p* < 0.05 for obese vs normal weight.

Tukey’s *post hoc* analyses revealed that obese children had significantly (*p* < 0.05) higher body fat percentages, SBP and DBP, as well as a significantly (*p* < 0.05) lower aerobic capacity (VO_2max_) than both normal weight and overweight children. In addition, there was a significant difference in salivary CRP concentration and salivary CRP flow rate between normal weight and obese children (*p* < 0.05). There were no significant differences between the normal versus overweight and overweight versus obese categories for salivary CRP concentration or secretion rate.

The non-log transformed salivary CRP concentrations ranged from 217.99 to 24562.94 pg/ml (median = 700.10 pg/min and interquartile range = 546.45–1372.14 pg/ml) and salivary CRP secretion rate ranged from 113.90 to 20694.28 pg/min (median = 683.41 pg/min and interquartile range = 500.26–1276.29 pg/min). These data were used in the binary logistic regression analysis (Table 2).

**Table 2.** Unadjusted And Adjusted Risk Factors For Elevated Salivary CRP In Children

*Risk factor*	*% of children with elevated salivary CRP and risk factor present (%)*	*% of children with elevated salivary CRP and risk factor absent (%)*	*Unadjusted odds ratio (95% CI)*	*Adjusted odds ratio (95% CI)*	p*-value*
Salivary CRP concentration					
VO_2max_ ≤ 50th percentile	35.3	12.3	3.9 (1.7–8.9)	3.9 (1.7–8.9)	0.001
BMI ≥ 85th percentile	30.6	17.8	2.0 (1.0–4.3)	1.0 (0.3–3.2)	0.944
Body fat (%) ≥ 25% for boys and ≥ 32% for girls	34.3	17.0	2.5 (1.2–5.3)	1.7 (0.7–3.7)	0.221
Salivary CRP secretion rate					
VO_2max_ ≤ 50th percentile	34.5	13.5	3.4 (1.2–7.5)	2.7 (1.2–6.1)	0.02
BMI ≥ 85th percentile	34.1	13.7	3.3 (1.5–7.3)	2.5 (1.1–5.9)	0.03
Body fat (%) ≥ 25% for boys and ≥ 32% for girls	37.1	14.8	3.4 (1.6–7.3)	1.8 (0.6–5.5)	0.40

Table 2 describes the magnitude of the relationship (unadjusted and adjusted odds ratios) between the selected risk factors (BMI ≥ 85th percentile; body fat % ≥ 25% for boys and ≥ 32% for girls; VO_2max_ ≤ 50th percentile) and elevated (defined as ≥ 75th percentile) salivary CRP concentration and secretion rate, calculated as ≥ 1372.14 pg/ml and ≥ 1276.39 pg/min, respectively. Only a poor CRF (≤ 50th percentile) was independently associated with elevated salivary CRP concentrations (≥ 75th percentile, ≥ 1372.14 pg/ml) (OR 3.9, 95% CI: 1.7–8.9, *p* = 0.001). However, both poor CRF (OR 2.7, 95% CI: 1.2–6.1, *p* = 0.02) and overweight/obesity (BMI ≥ 85th percentile) (OR 2.5, 95% CI: 1.1–5.9, *p* = 0.03) were independent predictors of elevated salivary CRP secretion rates (≥ 75th percentile, ≥ 1276.39 pg/ml).

## Discussion

The present study aimed to determine the relationship between salivary CRP, a non-invasive alternative marker of systemic inflammation, and body composition and/or CRF in black South African children. The main findings of this study were that poor CRF was an independent predictor of elevated salivary CRP concentration, while poor CRF and being overweight/obese were independent predictors of elevated salivary CRP secretion rate. The study suggests that both CRF and body composition are associated with elevated inflammatory status in black South African children and provides initial evidence that the measurement of salivary CRP may be a useful tool for examining the health status of children.

The study found that a normal BMI does not induce a state of inflammation as reflected by significantly lower salivary CRP concentration and secretion rates in normal weight compared to obese children. In addition, a normal weight was associated with a higher aerobic capacity (VO_2max_) and lower SBP and DBP compared to obesity.

The binary logistic regression analysis indicated the magnitude of the relationship between the risk factors and inflammatory status of the children. The results demonstrated that the odds ratio for the association between poor CRF and elevated salivary CRP concentration in children was 3.9. However, the odds ratio for elevated salivary CRP secretion rate if the children had both poor CRF and were overweight/obese was 6.8 (2.7 × 2.5).

These results are similar to previous research examining the relationship between serum CRP, CRF and body composition in Canadian children and young adults (6–24 years),^23^ and black South African children (13–18 years).^13^ The results for salivary CRP concentration is supported by a recent study demonstrating an inverse relationship between serum CRP levels and CRF that was independent of body composition and fat distribution.^7^ In addition, serum CRP concentrations in the overweight and obese categories were significantly higher in the low CRF category.^7^ The results from the present study therefore seem to support the hypothesis that regular exercise or physical activity and/or decreased visceral fat mass decreases low-grade inflammation.^1^,^6^,^11^

The present results, together with the recent finding that salivary CRP is a good measure of discrimination for the clinically relevant serum CRP cut-off point in adults,^7^,^16^ suggest that salivary CRP could be used as an alternative marker of cardiovascular or chronic disease risk in children. However, research is required to establish the relevance and cost effectiveness of this inflammatory marker in clinical practice.

In future, the determination of a clinically relevant index of salivary CRP may facilitate the prospective measurements of CRP levels in large epidemiological samples and contribute to understanding the mechanisms by which inflammation may be associated with the development of chronic disease in children. In addition, a clinically relevant index of salivary CRP may also be integrated into a broader multi-systemic non-invasive characterisation of chronic disease risk in children, together with other salivary biomarkers (e.g. cortisol, α-amylase, dehydroepiandrosterone) and BMI, CRF and percentage body fat.

There are a number of limitations that may have influenced the salivary CRP measurements in our study. Although salivary flow rate was controlled for, blood contamination, saliva pH, and health condition of the children’s gums were not determined using standardised methods. Specifically, a competitive immunoassay to quantify the presence of transferrin should be used to determine blood contamination of saliva,^7^ while saliva pH should be determined using calibrators with a known pH.^7^ It is possible that the determination of salivary CRP could be contaminated by small blood leakages or crevicular fluid overflow due to micro-injuries or in participants with poor oral health, as well as local inflammatory processes, which may elevate salivary CRP levels.^7^

However, in an attempt to reduce the impact of these confounding variables, clear instructions were provided to the parents as well as the participants regarding brushing teeth as well as dietary and hydration practices prior to saliva collection. It is recommended that future studies should include a complete oral health examination as part of the participant’s health assessment to eliminate the possibility that bucco-dental features trigger high CRP levels in saliva in the absence of elevations in the serum.^7^

Related to the issue of oral health in the children and the possible existence of periodontitis, a link was recently found between periodontitis, systemic inflammation and CVD.^28^ Specifically, it was found that intensive periodontal therapy reduced local as well as systemic reductions in inflammation, which correlated with an improvement in endothelial functioning.^28^ This finding suggests that children with a high CRF and normal weight may still have elevated serum/salivary CRP levels (systemic/local inflammation) and be at risk for developing chronic disease, if they have poor oral health.

## Conclusion

The study provides initial support for a possible association between poor CRF and/or overweight/obesity and inflammatory status in children, based on elevated salivary CRP levels. Saliva sampling is non-invasive, stress-free, can easily be performed in a participant’s natural setting and can be repeated over time. Furthermore, saliva collection has considerable economic and logistic advantages over venipuncture because it does not require immediate manipulations, access to specialised laboratory equipment and qualified personnel.

Replication of the study with larger samples is required together with longitudinal follow up of clinical outcomes. This may contribute to a better understanding of the pathways mediating the development and treatment of inflammation and chronic disease in children and subsequently adults.
